# Simultaneous high-definition transcranial direct current stimulation and robot-assisted gait training in stroke patients

**DOI:** 10.1038/s41598-024-53482-6

**Published:** 2024-02-23

**Authors:** Eunmi Kim, Gihyoun Lee, Jungsoo Lee, Yun-Hee Kim

**Affiliations:** 1 Department of Physical and Rehabilitation Medicine, Sungkyunkwan University School of Medicine, Suwon, 16419 Republic of Korea; 2 Interdisciplinary Program of Biomedical Engineering, Chonnam National University, Yeosu, 59626 Republic of Korea; 3 School of Healthcare and Biomedical Engineering, Chonnam National University, Yeosu, 59626 Republic of Korea; 4 Department of Medical IT Convergence Engineering, Kumoh National Institute of Technology, Gumi, 39253 Republic of Korea; 5 Haeundae Sharing and Happiness Hospital, Busan, 48101 Republic of Korea

**Keywords:** Diseases, Neurological disorders

## Abstract

This study investigates whether simultaneous high-definition transcranial direct current stimulation (HD-tDCS) enhances the effects of robot-assisted gait training in stroke patients. Twenty-four participants were randomly allocated to either the robot-assisted gait training with real HD-tDCS group (real HD-tDCS group) or robot-assisted gait training with sham HD-tDCS group (sham HD-tDCS group). Over four weeks, both groups completed 10 sessions. The 10 Meter Walk Test, Timed Up and Go, Functional Ambulation Category, Functional Reach Test, Berg Balance Scale, Dynamic Gait Index, Fugl-Meyer Assessment, and Korean version of the Modified Barthel Index were conducted before, immediately after, and one month after the intervention. The real HD-tDCS group showed significant improvements in the 10 Meter Walk Test, Timed Up and Go, Functional Reach Test, and Berg Balance Scale immediately and one month after the intervention, compared with before the intervention. Significant improvements in the Dynamic Gait Index and Fugl-Meyer Assessment were also observed immediately after the intervention. The sham HD-tDCS group showed no significant improvements in any of the tests. Application of HD-tDCS during robot-assisted gait training has a positive effect on gait and physical function in chronic stroke patients, ensuring long-term training effects. Our results suggest the effectiveness of HD-tDCS as a complementary tool to enhance robotic gait rehabilitation therapy in chronic stroke patients.

## Introduction

Stroke is the second leading cause of death worldwide, and its incidence has increased as the population has aged^1^. Despite a variety of physiotherapeutic options for improving functional outcomes after stroke, more than 30% of stroke survivors cannot walk independently^2^. Gait disorders caused by motor impairments of the lower limbs greatly affect patient quality of life and ability to carry out activities of daily living (ADLs)^3^.

Various methods for enhancing neuroplasticity to improve physical recovery after stroke have been considered. In addition to conventional rehabilitation (i.e., physical, occupational, and speech therapy), several new approaches have emerged during the past two decades, including robot-assisted training, brain stimulation, virtual reality, and cell therapy^4^. However, those treatments have not always resulted in superior outcomes compared with conventional rehabilitation; novel combination treatments are needed to better activate neuroplasticity mechanisms and enhance therapeutic effects^5^.

Based on neuroplasticity and motor learning, robot-assisted gait training (RAGT) provides intensive, repetitive, and accurate kinematic feedback and symmetric gait practice to induce adaptive modification and reorganization of neural connections and networks, maximizing recovery and functional outcomes^6^. A recently updated Cochrane review of 62 trials involving 2440 participants found that RAGT combined with physiotherapy was most beneficial for patients in the first three months after stroke and for those who were unable to walk^7^. Recovery capacity after stroke decreases over time, with maximum recovery occurring within the first 6 months after stroke^8^, and RAGT provides more improvement than conventional physical therapy in restoring gait ability, measured in terms of gait speed, balance, and motor functions, in subacute stroke patients^9–11^. Some studies have shown that RAGT improves balance and gait abilities by inducing functional improvements in the lower extremities not only in subacute stroke patients, but also in chronic stroke patients (more than six months after stroke)^12,13^. Although RAGT has the potential to improve gait ability in stroke patients, not all chronic stroke patients achieve satisfactory recovery^14^. Additional methods to enhance the effects of RAGT are needed.

Transcranial direct current stimulation (tDCS) might enhance the beneficial neuroplasticity effects of post-stroke rehabilitation^15^. By applying a low-intensity electrical current (e.g., 1–2 mA) to the scalp in the target cortical area, anodal tDCS can increase and cathodal tDCS can decrease the cortical excitability of the motor area^16^. Applying tDCS to the primary motor cortex (M1), which controls movement of the upper and lower extremities, increases its activity, improving motor learning in both healthy individuals and post-stroke patients^17^. Because tDCS is considered safe and is highly portable, it can easily be used simultaneously with other rehabilitation therapies, such as gait training^18^. Intensive upper extremity motor training in combination with tDCS has been shown to improve upper extremity motor function after stroke^19–22^. Although few studies have investigated the efficacy of tDCS in assisting the recovery of lower extremity function after stroke, the method has been shown to improve muscle strength in the lower extremities^23–25^.

M1 is a brain region located in the dorsal part of the frontal lobe in humans. Because the M1 cortical area that controls the lower extremities is in the medial part of the precentral gyrus, deep within the interhemispheric fissure, targeting conventional tDCS to this area is more challenging than targeting the upper extremities^26^. High-definition tDCS (HD-tDCS), which uses smaller high-definition electrodes to improve stimulation of the target area, has been proposed^27^. HD-tDCS can reportedly target deeper brain structures than tDCS^28^ and can be configured with several channels and multiple small electrodes in a variety of montages to guide current flow. It is conceivable that a combination of HD-tDCS and RAGT could effectively stimulate the more deeply located M1 leg area and enhance the effects of this combination therapy, compared with conventional tDCS, in chronic stroke patients^29,30^.

Our purpose in this study was to investigate whether HD-tDCS could enhance the training effects of RAGT on gait and physical function in chronic stroke patients with gait disorder. We hypothesized that combining HD-tDCS with RAGT would significantly enhance gait and physical function in chronic stroke patients, compared with RAGT only, and that this effect would be present for at least one month after the intervention.

## Results

**Figure 1:**
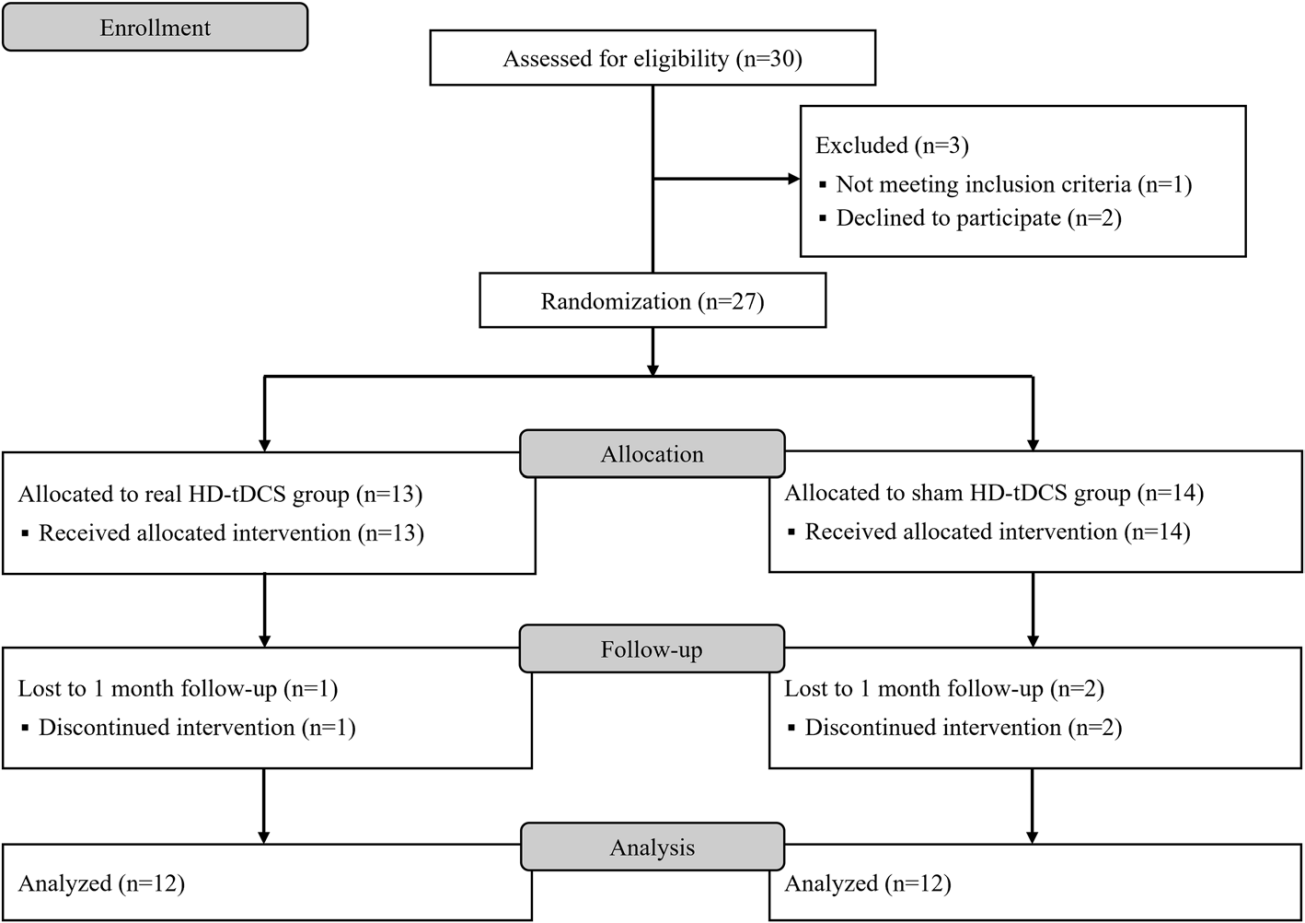
Study flow diagram. Real HD-tDCS group, robot-assisted gait training with real high-definition transcranial direct current stimulation; Sham HD-tDCS group, robot-assisted gait training with sham high-definition transcranial direct current stimulation.

Twenty-seven participants who met the inclusion criteria were randomly assigned to the RAGT with real HD-tDCS (Real HD-tDCS) group or RAGT with sham HD-tDCS (Sham HD-tDCS) group and received their allocated interventions (Fig. 1). The final analysis included 24 participants, excluding three who discontinued the intervention for personal reasons (two participants in the real HD-tDCS group and one in the sham HD-tDCS group). The two groups had no significant differences in baseline characteristics before the intervention (Pre) (Table 1).

**Table 1:** Baseline characteristics.

Characteristics	Total (n = 24)	Real HD-tDCS group (n = 12)	Sham HD-tDCS group (n = 12)
Age (years)	60.54 (13.90)	62.58 (11.22)	58.50 (16.41)
Sex (male/female)	15/9	7/5	8/4
Time since onset (month)	52.50 (45.04)	59.17 (58.92)	45.83 (25.94)
Affected side (right/left)	15/9	7/5	8/4
Stroke type (infarction/hemorrhage)	15/9	6/6	9/3
Height (cm)	164.30 (9.64)	163.83 (8.87)	164.77 (10.72)
Weight (kg)	67.42 (10.74)	67.32 (8.23)	67.52 (13.17)
BMI (kg/m^2^)	24.91 (2.91)	25.07 (2.21)	24.74 (3.57)
FAC	3.29 (0.69)	3.50 (0.52)	3.08 (0.79)
K-MMSE	27.96 (2.46)	27.83 (2.92)	28.08 (2.02)

Continuous values are presented as means (standard deviations).

Real HD-tDCS group, robot-assisted gait training with real high-definition transcranial direct current stimulation; Sham HD-tDCS group, robot-assisted gait training with sham high-definition transcranial direct current stimulation; BMI, body mass index; FAC, Functional Ambulation Category; K-MMSE, Korea-Mini Mental State Examination.

**Figure 2:**
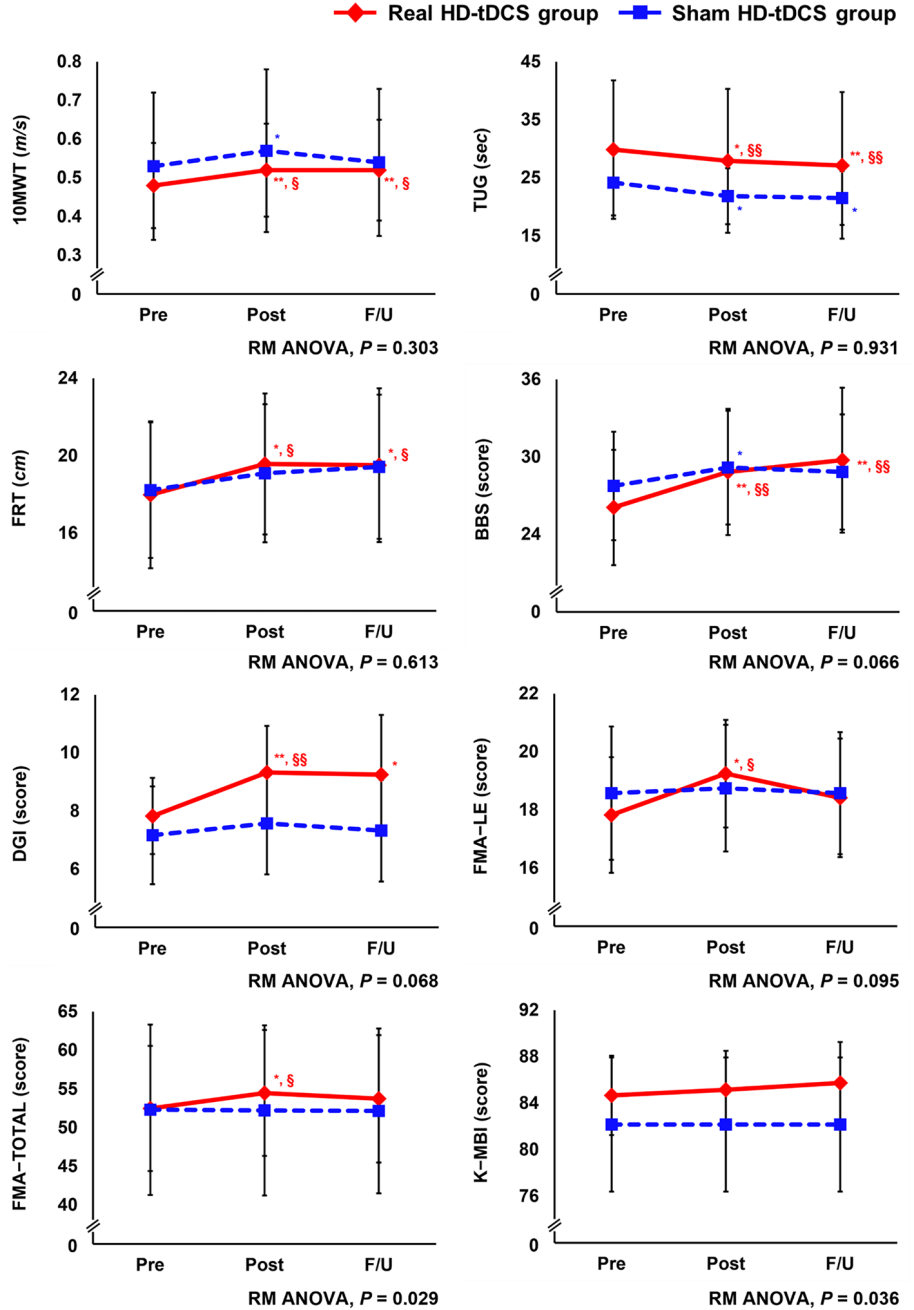
Effect of robot-assisted gait training with high-definition transcranial direct current stimulation on gait and physical function. Real HD-tDCS group, robot-assisted gait training with real high-definition transcranial direct current stimulation; Sham HD-tDCS group, robot-assisted gait training with sham high-definition transcranial direct current stimulation; 10MWT; 10 Meter Walk Test; TUG, Timed Up and Go; FRT, Functional Reach Test; BBS, Berg Balance Scale; DGI, Dynamic Gait Index; FMA, Fugl-Meyer Assessment; LE, lower extremity; K-MBI, Korean Version of the Modified Barthel Index; Pre, pre-intervention; Post, post-intervention; F/U, one-month follow-up; RM ANOVA, repeated measures analysis of variance. ^*^Significant change compared with Pre (*P* < 0.05); ^**^Significant change compared with Pre (*P* < 0.01) using the Wilcoxon signed rank test. ^§^Significant change compared with Pre (*P* < 0.05); ^§§^Significant change compared with Pre (*P* < 0.01) using Bonferroni’s post hoc analysis of repeated measures analysis of variance.

Physical functions in the real and sham HD-tDCS groups at Pre, immediately after the intervention (Post), and at the follow-up (F/U) visit one month after the intervention are illustrated in Fig. 2; significance was determined by the Wilcoxon signed rank test or Bonferroni’s post hoc analysis of repeated measures analysis of variance (RM ANOVA). Specific statistical values for the 10 Meter Walk Test (10MWT), Timed Up and Go (TUG), Functional Ambulation Category (FAC), Functional Reach Test (FRT), Berg Balance Scale (BBS), Dynamic Gait Index (DGI), Fugl-Meyer Assessment (FMA), and Korean version of the Modified Barthel Index (K-MBI) outcome measures at the Pre, Post, and F/U time points are presented in Supplementary Table S1 and Supplementary Table S2. Lower extremity FMA scores (FMA–LE) and total upper extremity and lower extremity scores (FMA–TOTAL) for the affected side were calculated. The groups did not differ in gait, balance, motor function, or ADL performance at the Pre, Post, and F/U time points.

In the real HD-tDCS group, 10MWT (*Z* = − 2.707, *P* = 0.007), TUG (*Z* = − 2.590, *P* = 0.010), FRT (*Z* = − 2.448, *P* = 0.014), BBS (*Z* = − 2.825, *P* = 0.005), DGI (*Z* = − 2.630*, P* = 0.009), FMA-LE (*Z* = − 2.539, *P* = 0.011), and FMA-TOTAL (*Z* = − 2.386*, P* = 0.017) scores all improved significantly from the Pre to Post time points. In the sham HD-tDCS group, statistically significant changes from the Pre to Post time points were found in 10MWT (*Z* = − 2.040, *P* = 0.041), TUG (*Z* = − 2.197,* P* = 0.028), and BBS (*Z* = − 2.198*, P* = 0.028). Significant improvement from Pre to F/U in the 10MWT (*Z* = − 2.732*, P* = 0.006), TUG *(Z* = − 2.746, *P* = 0.006), FRT (*Z* = − 2.446*, P* = 0.014), BBS (*Z* = − 2.706*, P* = 0.007), and DGI (*Z* = − 2.056, *P* = 0.040) was noted in the real HD-tDCS group, but the sham HD-tDCS group showed significant changes from Pre to F/U only in TUG *(Z* = − 2.040*, P* = 0.041).

Bonferroni’s post hoc analysis revealed that the 10MWT (95% CI [− 0.077, − 0.005], *P* = 0.026), TUG (95% CI [0.506, 3.392], *P* = 0.009), FRT (95% CI [− 3.020, − 0.162], *P* = 0.029), BBS (95% CI [− 4.623, − 0.877], *P* = 0.005), DGI (95% CI [− 2.570, − 0.430], *P* = 0.007), FMA-LE (95% CI [− 2.642, − 0.191], *P* = 0.023), and FMA-TOTAL (95% CI [-3.837, -0.163], *P* = 0.032) scores in the real HD-tDCS group changed significantly from Pre to Post. Furthermore, 10MWT (95% CI [− 0.070, − 0.002], *P* = 0.039), TUG (95% CI [0.770, 4.687], *P* = 0.007), FRT (95% CI [2.880, − 0.193], *P* = 0.025), and BBS (95% CI [− 5.978, − 1.355], *P* = 0.003) improved significantly from Pre to F/U in the real HD-tDCS group. On the other hand, there were no significant improvements in these scores in the sham HD-tDCS group.

In addition, a significant time × group interaction was found in FMA − TOTAL in the real HD-tDCS group, indicating improvements compared with the sham HD-tDCS group (*F* = 4.199*, P* = 0.029). RM ANOVA revealed a significant time × group interaction in K-MBI (*F* = 3.591*, P* = 0.036) in the real HD-tDCS group, indicating improvement in this measure compared with the sham HD-tDCS group across all time points.

After each intervention session, participants were asked to rate their pain and discomfort. The pain scale ranged from 0 to 10, but the highest supplied response was 3. Among the real HD-tDCS group, 41.7% of participants did not feel any pain (score of 0), 16.7% answered that they felt almost no pain (score of 1), 16.7% reported experiencing some pain (score of 2), and 25% had some pain that was quickly forgotten (score of 3). The scale for rating discomfort during the intervention ranged from 0 to 4; 50% of participants in the real HD-tDCS group felt no discomfort (score of 0), and the remaining 50% answered that the discomfort was negligible (score of 1). No participants reported pain or discomfort during the intervention in the sham HD-tDCS group.

## Discussion

Our purpose in this study was to investigate whether simultaneous application of HD-tDCS and RAGT would enhance the training effects of RAGT in chronic stroke patients. Our results show improvements in gait and balance function (10MWT, TUG, and BBS) in both groups. Significant improvements in gait, balance, and motor functions (10MWT, TUG, FRT, BBS, DGI, FMA–LE, and FMA–TOTAL) were found in the real HD-tDCS group, and the improvements in gait and balance function (10MWT, TUG, FRT, BBS, and DGI) in the real HD-tDCS group were maintained for at least one month after the intervention.

A systematic review found that subacute stroke patients who are unable to walk independently received more beneficial effects from RAGT than chronic stroke patients^7^. However, most patients included in that review were already capable of independent gait at the start of the study^7^, which might have diluted the benefits of RAGT for chronic stroke patients. Therefore, there remains the possibility that RAGT may improve gait ability in chronic stroke patients. Choi et al. (2022) reported improvements in the functional gait and balance ability of chronic stroke patients who received RAGT with body weight support five times a week for six weeks^31^. Even when no statistically significant changes were found between the effects of RAGT and conventional physical therapy in chronic stroke patients, training-related improvements were reported in the RAGT group in terms of balance, as well as physical function, spatiotemporal gait parameters, and gait symmetry^32,33^. Our findings indicate improvements in gait and balance function in both groups following a RAGT intervention three times a week for four weeks. The increases in gait and balance function in both groups after the intervention might be due to the advantages of RAGT over conventional physical therapy: a larger number of steps can be practiced per session^34^, symmetrical gait can be facilitated^33^, and paretic leg step length symmetry is fostered^32^. However, the effects of RAGT in chronic stroke patients are still controversial. Therefore, diversification of the training protocol and tDCS application can be considered to enhance the effectiveness of RAGT in chronic stroke patients.

Three previous studies evaluated the effects of applying real and sham tDCS with RAGT in chronic stroke patients. Geroin et al.^35^ applied 1.5 mA anodal tDCS during the first 7 min of RAGT and reported that tDCS provided no additional benefit to RAGT in chronic stroke patients. Danzl et al.^36^ set the intensity of tDCS to 2 mA, applied it for 20 min, and performed RAGT after each tDCS session, repeating that intervention three times a week for four weeks. They reported a significant improvement in FAC scores after RAGT with real tDCS, compared with before the intervention^36^. Seo et al.^37^ applied tDCS to the leg motor cortex for 20 min every weekday for 2 weeks, performing RAGT for 45 min after each session. They found statistical improvements in the FAC and 6-Minute Walk Test in the real tDCS group at the one-month follow-up after the intervention^37^. These studies generally found improvements in gait, balance, and motor abilities, but the results were not consistent across measures such as 10MWT, BBS, and FMA. Differences in tDCS intensity, time, and stimulation area might explain the different results among the previous studies.

In this study, we found significant improvements in gait, balance, and motor functions after real HD-tDCS application in combination with RAGT. These results suggests that the combined application of HD-tDCS and RAGT might facilitate the training effects of RAGT by increasing its consistency in chronic stroke patients^38^. In our study, there was a statistically significant improvement in 10MWT after the intervention in the real HD-tDCS group, consistent with the small significant changes reported by Perera et al.^39^. The sample size of our study was calculated using minimal clinically important difference (MCID) of 10MWT obtained from the previous study^39^. Although the change in 10MWT in our study was not higher than the MCID used for sample size calculation, it was statistically significant and met the small meaningful changes in stroke survivors. Furthermore, the real HD-tDCS group maintained gait and balance function until the F/U time point, thus the combination of RAGT and HD-tDCS appeared to give long-term positive effects in these patients^40,41^. Differences in the number of interventions, duration, order of applying RAGT and HD-tDCS, intensity, target area of stimulation, and montage of the HD-tDCS electrodes explained differences between the results of this study and those of previous studies. Nevertheless, the montage of HD-tDCS electrodes used in this study can be applied for enhancing the effect of RAGT on gait and balance function of chronic stroke patients in future.

Rehabilitation of physical function among stroke patients is important because a decline in these functions increases the risk of falls during walking and reduces quality of life^42^. A study investigating a training protocol for stroke patients that combined RAGT with individualized training by a therapist reported that the protocol significantly improved mobility, ADLs, and quality of life^43^. tDCS also improves ADL performance and physical function in stroke patients^44^. Consistent with previous findings, we found significant time × group interactions in improved ADL performance and motor function in the real HD-tDCS group compared with the sham HD-tDCS group. These results suggest that the combination of HD-tDCS and RAGT has greater benefit for motor and ADL function than RAGT alone. In addition, combining RAGT with HD-tDCS could lead to changes in neuroplasticity that promote physical recovery and enhance subsequent spontaneous activities, such as ADLs, by increasing cortical activity^45^.

This study had several limitations. First, it was an exploratory clinical trial with a small number of participants; further studies using a larger stroke population are needed to validate the findings. Second, we did not include a true control group (conventional physical therapy or no treatment) that received neither HD-tDCS nor RAGT. Third, lesion sites, such as cortical and subcortical lesions, and stroke types were not considered when recruiting study participants (Supplementary Table S3), even though the effects of HD-tDCS can be influenced by lesion location and size. An analysis of effects according to stroke type and lesion location will provide further guidance on how best to combine HD-tDCS and RAGT. Despite those limitations, most participants reported no or negligible pain or discomfort, confirming that the HD-tDCS device was safely applied for 30 min during RAGT.

Our results demonstrate that simultaneous application of HD-tDCS and RAGT had a larger positive effect on the gait and physical function of chronic stroke patients than RAGT alone. Combining RAGT with HD-tDCS ensured that training effects lasted for up to one month. HD-tDCS can be used as a complementary tool to enhance robotic gait rehabilitation therapy in chronic stroke patients after larger confirmatory studies have verified our findings.

## Methods

### Participants

This was a single-center, double-blind, randomized, prospective study. Participants were assigned to one of two groups: (1) Real HD-tDCS group or (2) Sham HD-tDCS group. The inclusion criteria were (1) chronic phase of stroke (at least six months after stroke onset), (2) age 19 to 79 years, and (3) gait disorder with a FAC score of 1 to 4. The exclusion criteria were (1) a history of serious neurological disease other than stroke (e.g., Parkinson’s disease), (2) severe cognitive deficits (a Korean-Mini Mental State Examination (K-MMSE) score ≤ 10), (3) a history of serious mental illness (e.g., schizophrenia or bipolar disorder), (4) a metallic object in the skull, (5) a history of epilepsy, (6) current pregnancy or lactation, (7) an implantable medical device (e.g., a pacemaker), and (8) any dermatological problem that prevented attachment of the stimulation electrodes. The baseline characteristics of the study participants are summarized in Table 1. The number of participants was calculated using Lehr’s formula^46^, which has a power of 80% and an effective level of 5%. This was calculated with the MCID (0.14 m/s)^39^ and a standard deviation of 0.12^47^ in the 10MWT, which was the primary outcome of this study. The calculated number of participants needed in each group was 12, for a total of 24 participants.

Written informed consent was obtained from all participants before the experiments in accordance with the Declaration of Helsinki, and the protocol was approved by the Institutional Review Board of Samsung Medical Center (IRB no. 2021-06-131, 12/08/2021). The study protocol and consent form were also reviewed and approved by the Korean Food and Drug Administration (No. 1227, 07/07/2021), and the study was registered with ClinicalTrials.gov (NCT04985864, 16/08/2021).

### Experimental protocol

**Figure 3:**
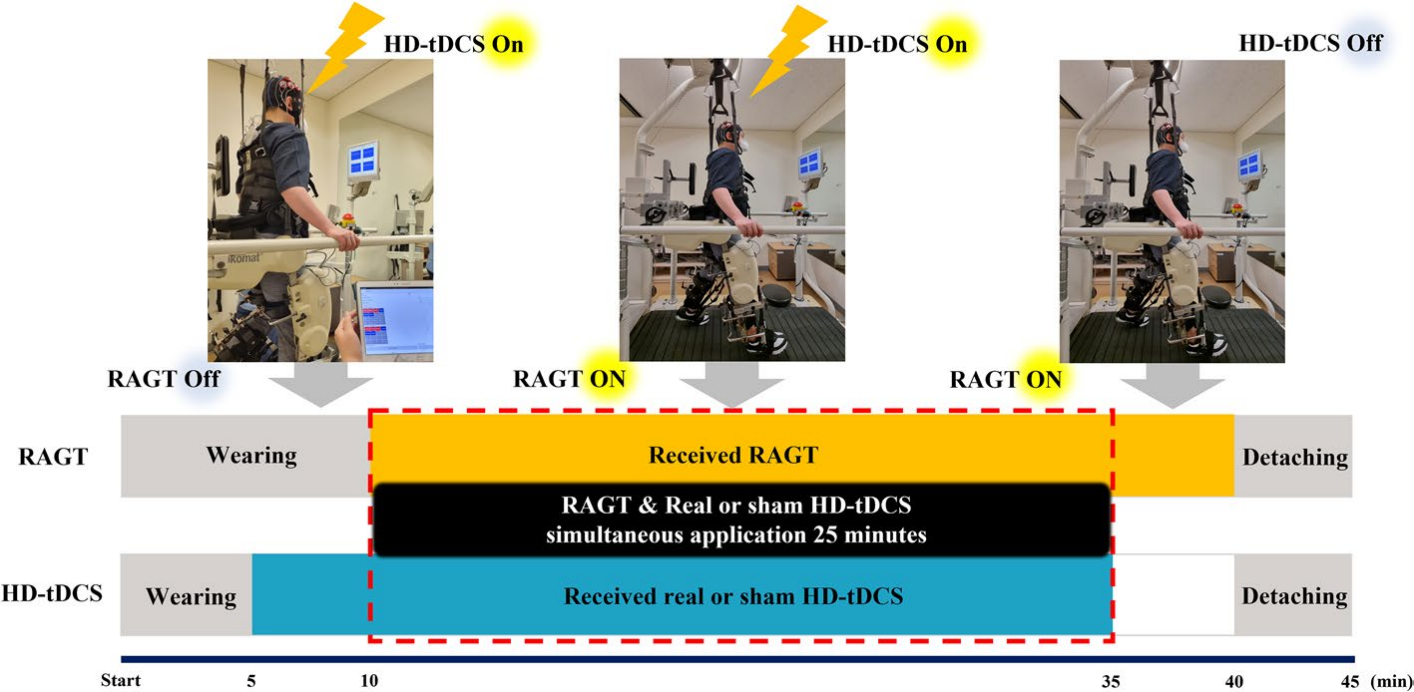
Intervention protocol.

Participant age, sex, height, weight, stroke type, time since onset, FAC, and K-MMSE scores were recorded and evaluated to determine whether participants met the inclusion criteria. Eligible participants were randomly assigned to either the real HD-tDCS or sham HD-tDCS group using a randomization table. All participants underwent 10 sessions over four consecutive weeks (approximately three times a week). The duration of each intervention session was 45 min (Fig. 3). The intervention protocol was as follows: (1) participants were fitted with a cap equipped with HD-tDCS electrodes and wore a RAGT for 5 min; (2) while wearing the RAGT, participants received real or sham HD-tDCS for 5 min; (3) participants received RAGT and simultaneous real or sham HD-tDCS for 25 min; (4) for the next 5 min, participants received only RAGT without real or sham HD-tDCS; and (5) the RAGT and HD-tDCS were detached. Our protocol was designed to activate the central nervous system through HD-tDCS for 5 min before RAGT and to provide simultaneous peripheral nervous system stimulation during RAGT for a sufficient period of motor learning without fatigue^48^. This study was double-blind for both evaluators and participants. The two evaluators who measured the physical functions of the participants were not involved in the interventions and were unaware of the assigned groups. All outcome measures were evaluated at Pre, Post, and F/U.

### Robot-assisted gait training

The Lokomat® (Hocoma AG, Zurich, Switzerland) is a RAGT device that uses a harness to support patients while their legs are attached to robotic limbs that control the movements of the bilateral hip, knee, and ankle joints to replicate normal gait patterns on a treadmill. Movement of the participant’s lower extremities was based on a pre-programmed normal gait pattern and allowed a bilaterally symmetrical gait pattern as they attempted to walk on the treadmill. The Lokomat® protocol for gait training offers intensive, task-oriented, repetitive training for gait restoration in stroke patients^32^. During Lokomat® treatment, an experienced therapist was able to adjust the body weight support, gait speed, and hip and knee joint angles to match each participant’s physical function. Speed was increased from 1.2 km/h up to the maximum speed to which each participant could adapt; the selected speed ranged from 1.4 to 2.0 km/h. The therapist gradually reduced the amount of body weight support from 40 to 0% based on the participant’s gait pattern, and the guidance force was set from 100 to 0% depending on the participant’s tolerance. Participants were required to participate more actively as the guidance force was reduced, and the therapist encouraged participants to walk actively. All parameters were adjusted progressively according to improvement in the participant’s gait function, and they were individually adjusted for each session. Participants underwent training for 30 min.

### High-definition transcranial direct current stimulation

**Figure 4:**
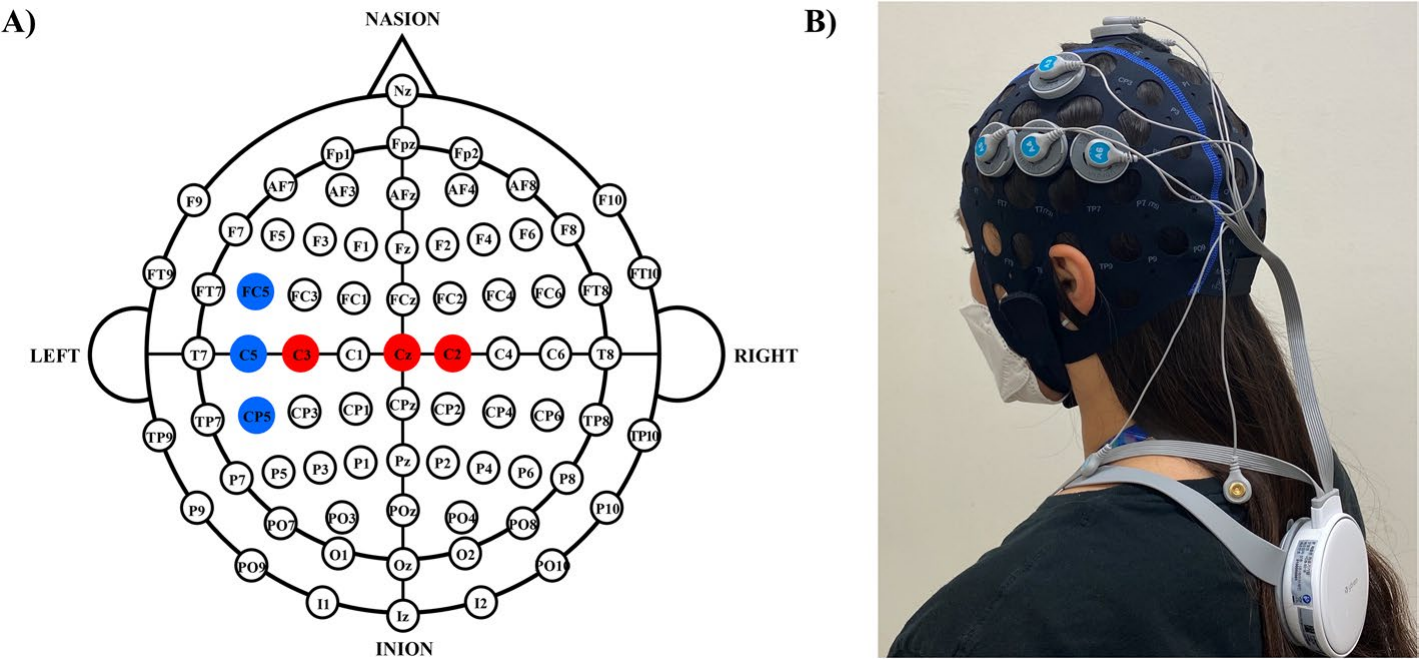
Electrode montage for high-definition transcranial direct current stimulation. (**A**) The position and intensity of the anodes (Cz = 1 mA; C2 = 0.7 mA; C3 = 0.3 mA) and cathodes (C5 =  − 1 mA; FC5 =  − 0.5 mA; CP5 =  − 0.5 mA) in participants with a lesion of the left hemisphere. For participants with a lesion of the right hemisphere, electrodes were placed in the corresponding positions on the right side of the head. (**B**) Electrodes placed according to the selected montage in a participant with a left hemisphere lesion.

The HD-tDCS used in this study was the YDS-501B® device developed by YBrain (YBrain Inc., Pangyo, Republic of Korea), which can accommodate 2 to 16 electrodes (2 cm in diameter). Using Neurophet tES Lab® brain stimulation simulation software (Neurophet Inc., Seoul, Republic of Korea) with standard MRI images of the brain, the optimal electrode position to provide the maximum current to the leg motor area in M1 was selected. HD-tDCS was applied at 2 mA for 30 min to enhance the effects of RAGT in chronic stroke patients^49^. The default settings were six electrodes, maximum current strength of 1 mA per electrode, and a total current strength of 2 mA. Based on the 10–20 system, the position and strength of the anodes and cathodes were set as follows: (1) anode: Cz = 1 mA, C2 = 0.7 mA, C3 = 0.3 mA; (2) cathode: C5 =  − 1 mA; FC5 =  − 0.5 mA; CP5 =  − 0.5 mA. In participants with a lesion of the left hemisphere, the cathode was placed on the left side to ensure that current flowed to the left side; for participants with a lesion of the right hemisphere, cathodes were placed on the right. The position of the electrodes was the same in both groups, and the HD-tDCS device was set to 0 mA intensity of stimulation for 30 min for the sham group (Fig. 4). To ensure that participants were unaware of their group assignment, they wore the HD-tDCS device with the power on and watched the HD-tDCS stimulation software program being set up.

### Physical function evaluation

To measure gait, balance, motor functions, and ADL performance, the 10MWT, TUG, FAC, FRT, BBS, DGI, FMA, and K-MBI were performed.

The 10MWT is widely used to determine the functional mobility, gait, and vestibular function of stroke patients^50^. The TUG test assesses mobility, balance, gait ability, and the risk of fall in stroke patients and evaluates the ability to maintain balance during gait movements^51^. The FAC is used to assess functional ambulation by determining the amount of assistance required by a participant when walking, regardless of whether aids are used^52^. FRT assesses a patient’s stability and static balance by measuring the maximum distance an individual can reach forward while standing in a fixed position^53^. BBS and DGI are used to objectively determine a stroke patient’s dynamic ability to remain safely balanced while performing predetermined tasks^54,55^. The FMA assesses motor function, sensation, balance, joint range of motion, and joint pain in patients with post-stroke hemiplegia^56^. The K-MBI is used among stroke patients to assess behavior relating to ADLs^57^.

### Visual analogue scale

A visual analogue scale (VAS) is a psychometric questionnaire used to evaluate subjective characteristics or attitudes that cannot be measured directly. A VAS was used to investigate participant pain and discomfort during the intervention and confirm the safety of the HD-tDCS device and intervention protocol. Each participant’s pain level was rated on a scale from 0 to 10, with 0 indicating no pain and 10 indicating extreme pain. The discomfort level was determined on a scale from 0 to 4, with 0 indicating no discomfort at all and 4 indicating that the patient was very uncomfortable. Each participant’s pain and discomfort were confirmed through a questionnaire after each intervention session.

### Statistical analysis

All data were analyzed using SPSS software version 25.0 (IBM Corp., Armonk, NY, USA). For all tests, the level of statistical significance was set to 0.05. Baseline characteristics of participants were compared between groups using the independent t-test or Mann–Whitney U test for continuous variables and the chi-square test for categorical variables. To evaluate the effects of the intervention, the Wilcoxon signed rank test was used to compare outcome measures between time points within groups. Changes between groups at each time-point were analyzed using Bonferroni’s post hoc analysis of RM ANOVA. Time × group interactions were examined using RM ANOVA of the real and sham HD-tDCS groups and all three time points (Pre, Post, and F/U).

### Ethics approval and consent to participate

All participants recruited through Samsung Medical Center provided informed consent before participating in the study. Written informed consent was obtained from all participants before the experiments, in accordance with the Declaration of Helsinki, and this study protocol was approved by the ethics committee of the Samsung Medical Center Institutional Review Board (IRB no. 2021-06-131, 12/08/2021). The study protocol and consent form were also reviewed and approved by the Korean Food and Drug Administration (No. 1227, 07/07/2021), and the study was registered with ClinicalTrials.gov (NCT04985864, 16/08/2021).

### Supplementary Information

Supplementary Tables.

## Data Availability

The datasets used and/or analyzed during the current study are available from the corresponding authors upon reasonable request.
