# Supplementary material for: Simultaneous high-definition transcranial direct current stimulation and robot-assisted gait training in stroke patients
Source: Sci Rep. 2024 Feb 23;14:4483. doi: 10.1038/s41598-024-53482-6 (PMC10891044; doi:10.1038/s41598-024-53482-6)
Supplement: Supplementary file 1 — Supplementary Tables. [file 41598_2024_53482_MOESM1_ESM.docx]

**Supplementary Table S1.** Changes in gait and physical function after robot-assisted gait training with high-definition transcranial direct current stimulation, with significance assessed using the Wilcoxon signed rank test.

|  | Real HD-tDCS group | | | | | | | | | | | | | |  | Sham HD-tDCS group | | | | | | | | | | | | | | |
| --- | --- | --- | --- | --- | --- | --- | --- | --- | --- | --- | --- | --- | --- | --- | --- | --- | --- | --- | --- | --- | --- | --- | --- | --- | --- | --- | --- | --- | --- | --- |
|  | Pre | Post | F/U |  | Pre-Post | | | Pre-F/U | | | Post-F/U | | | |  | Pre | Post | F/U |  | Pre-Post | | | Pre-F/U | | | Post-F/U | | | |  |
|  |  |  |  |  | Z | *P* | ES | Z | *P* | ES | | Z | *P* | ES |  |  |  |  |  | Z | *P* | ES | Z | *P* | ES | | Z | *P* | ES |  |
| 10MWT (*m/s*) | 0.48 (0.22) | 0.52 (0.24)^**^ | 0.52 (0.25)^**^ |  | -2.707^a^ | 0.007 | 0.781 | -2.732 ^a^ | 0.006 | 0.789 | | -0.784^b^ | 0.433 | 0.226 |  | 0.53 (0.38) | 0.57 (0.43)^*^ | 0.54 (0.39)^†^ |  | -2.040^a^ | 0.041 | 0.589 | -0.554^a^ | 0.580 | 0.160 | | -2.490^b^ | 0.013 | 0.719 |  |
| TUG (*s*) | 29.87 (23.82) | 27.92 (24.77)^*^ | 27.14 (25.21)^**^ |  | -2.590^b^ | 0.010 | 0.748 | -2.746^b^ | 0.006 | 0.793 | | -1.098^b^ | 0.272 | 0.317 |  | 24.16 (11.28) | 21.84 (9.68)^*^ | 21.52 (9.28)^*^ |  | -2.197^b^ | 0.028 | 0.634 | -2.040^b^ | 0.041 | 0.589 | | -0.549^a^ | 0.583 | 0.158 |  |
| FAC | 3.42 (0.67) | 3.50 (0.52) | 3.58 (0.67) |  | -1.000^a^ | 0.317 | 0.289 | -1.414^a^ | 0.157 | 0.408 | | -1.000^a^ | 0.317 | 0.289 |  | 3.17 (0.72) | 3.42 (0.90) | 3.42 (0.90) |  | -1.732^a^ | 0.083 | 0.500 | -1.732^a^ | 0.083 | 0.500 | | 0.000^c^ | 1.000 | 0.000 |  |
| FRT (*cm*) | 17.99 (7.57) | 19.58 (7.28)^*^ | 19.52 (7.93)^*^ |  | -2.448^a^ | 0.014 | 0.707 | -2.446^a^ | 0.014 | 0.706 | | -0.362^b^ | 0.717 | 0.105 |  | 18.23 (6.99) | 19.10 (7.12) | 19.43 (7.43) |  | -1.248^a^ | 0.212 | 0.360 | -1.872^a^ | 0.061 | 0.540 | | -0.089^a^ | 0.929 | 0.026 |  |
| BBS | 26.08 (8.92) | 28.83 (9.78)^**^ | 29.75 (11.21)^**,^ |  | -2.825^a^ | 0.005 | 0.814 | -2.706^a^ | 0.007 | 0.781 | | -1.768^a^ | 0.077 | 0.510 |  | 27.75 (8.40) | 29.17 (8.81)^*^ | 28.83 (8.92) |  | -2.198^a^ | 0.028 | 0.635 | -1.259^a^ | 0.208 | 0.363 | | -1.027^b^ | 0.305 | 0.296 |  |
| DGI | 7.83 (2.62) | 9.33 (3.20)^**^ | 9.25 (4.11)^*^ |  | -2.630^a^ | 0.009 | 0.759 | -2.056^a^ | 0.040 | 0.594 | | -0.213^a^ | 0.832 | 0.061 |  | 7.17 (3.35) | 7.58 (3.42) | 7.33 (3.50) |  | -1.179^a^ | 0.238 | 0.340 | -0.486^a^ | 0.627 | 0.140 | | -0.632^b^ | 0.527 | 0.182 |  |
| FMA−LE | 17.83 (3.97) | 19.25 (3.70)^*^ | 18.42 (4.08) |  | -2.539^a^ | 0.011 | 00.733 | -1.204^a^ | 0.229 | 0.348 | | -1.559^b^ | 0.119 | 0.161 |  | 18.58 (4.58) | 18.75 (4.35) | 18.58 (4.19) |  | -0.333^a^ | 0.739 | 0.096 | -0.175^b^ | 0.861 | 0.051 | | -1.000^b^ | 0.317 | 0.289 |  |
| FMA  −TOTAL | 52.50 (16.20) | 54.50 (16.27)^*^ | 53.75 (16.43) |  | -2.386^a^ | 0.017 | 0.689 | -1.222^a^ | 0.222 | 0.353 | | -0.797^b^ | 0.426 | 0.230 |  | 52.33 (22.03) | 52.25 (22.00) | 52.17 (21.29) |  | -0.264^b^ | 0.792 | 0.076 | -0.302^b^ | 0.763 | 0.087 | | -0.180^b^ | 0.857 | 0.052 |  |
| K-MBI | 84.67 (6.81) | 85.17 (6.66) | 85.75 (7.02) |  | -1.414^a^ | 0.157 | 0.408 | -1.890^a^ | 0.059 | 0.546 | | -1.342^a^ | 0.180 | 0.387 |  | 82.17 (11.54) | 82.17 (11.54) | 82.17 (11.54) |  | 0.000^c^ | 1.000 | 0.000 | 0.000^c^ | 1.000 | 0.000 | | 0.000^c^ | 1.000 | 0.000 |  |

Continuous values are presented as means (standard deviations).

Real HD-tDCS group, robot-assisted gait training with real high-definition transcranial direct current stimulation; Sham HD-tDCS group, robot-assisted gait training with sham high-definition transcranial direct current stimulation; Pre, pre-intervention; Post, post-intervention; F/U, one-month follow-up; 10MWT; 10 Meter Walk Test; TUG, Timed Up and Go; FAC, Functional Ambulation Category; FRT, Functional Reach Test; BBS, Berg Balance Scale; DGI, Dynamic Gait Index; FMA, Fugl-Meyer Assessment; LE, lower extremity; K-MBI, Korean Version of the Modified Barthel Index; ES, effect size.

^*^Significant change compared with Pre (*P* < 0.05); ^**^Significant change compared with Pre (*P* < 0.01); ^†^Significant change compared with Post (*P* < 0.05).

a, based on negative ranks; b, based on positive ranks; c, the sum of negative ranks equals the sum of positive ranks.

**Supplementary Table S2.** Time × group interactions and changes in gait and physical function after robot-assisted gait training with high-definition transcranial direct current stimulation analyzed using Bonferroni’s post hoc analysis of repeated measures analysis of variance.

|  | Real HD-tDCS group | | | | | | | | | |  | Sham HD-tDCS group | | | | | | | | | |  | Time × group  measures | |
| --- | --- | --- | --- | --- | --- | --- | --- | --- | --- | --- | --- | --- | --- | --- | --- | --- | --- | --- | --- | --- | --- | --- | --- | --- |
|  | Pre | Post | F/U |  | Pre-Post | | Pre-F/U | | Post-F/U | |  | Pre | Post | F/U |  | Pre-Post | | Pre-F/U | | Post-F/U | |  | F | *P* |
|  |  |  |  |  | *P* | 95% CI | *P* | 95% CI | *P* | 95% CI |  |  |  |  |  | *P* | 95% CI | *P* | 95% CI | *P* | 95% CI |  |  |  |
| 10MWT (*m/s*) | 0.48 (0.22) | 0.52 (0.24) | 0.52 (0.25) |  | 0.026 | -0.077~  -0.005 | 0.039 | -0.070~  -0.002 | 1.000 | -0.038~  0.049 |  | 0.53 (0.38) | 0.57 (0.43) | 0.54 (0.39) |  | 0.149 | -0.088~  0.011 | 1.000 | -0.053~  0.044 | 0.274 | -0.018~  0.086 |  | 1.229 | 0.303 |
| TUG (*s*) | 29.87 (23.82) | 27.92 (24.77) | 27.14 (25.21) |  | 0.009 | 0.506~  3.392 | 0.007 | 0.770~  4.687 | 0.497 | -0.700~  2.259 |  | 24.16 (11.28) | 21.84 (9.68) | 21.52 (9.28) |  | 0.122 | -0.503~  5.155 | 0.273 | -1.380~  6.668 | 1.000 | -2.497~  3.133 |  | 0.071 | 0.931 |
| FAC | 3.42 (0.67) | 3.50 (0.52) | 3.58 (0.67) |  | 1.000 | -0.318~  0.152 | 0.498 | -0.484~  0.150 | 1.000 | -0.318~  0.152 |  | 3.17 (0.72) | 3.42 (0.90) | 3.42 (0.90) |  | 0.246 | -0.618~  0.118 | 0.246 | -0.618~  0.118 | - | - |  | 1.453 | 0.245 |
| FRT (*cm*) | 17.99 (7.57) | 19.58 (7.28) | 19.52 (7.93) |  | 0.029 | -3.020~  -0.162 | 0.025 | -2.880~  -0.193 | 1.000 | -0.876~  0.985 |  | 18.23 (6.99) | 19.10 (7.12) | 19.43 (7.43) |  | 0.473 | -2.525~  0.771 | 0.215 | -2.921~  0.512 | 1.000 | -1.858~  1.204 |  | 0.496 | 0.613 |
| BBS | 26.08 (8.92) | 28.83 (9.78) | 29.75 (11.21) |  | 0.005 | -4.623~  -0.877 | 0.003 | -5.978~  -1.355 | 0.281 | -2.325~  0.492 |  | 27.75 (8.40) | 29.17 (8.81) | 28.83 (8.92) |  | 0.100 | -3.062 ~  0.228 | 0.452 | -3.061~  0.894 | 1.000 | -0.607~  1.273 |  | 3.095 | 0.066 |
| DGI | 7.83 (2.62) | 9.33 (3.20) | 9.25 (4.11) |  | 0.007 | -2.570~  -0.430 | 0.091 | -3.025~  0.191 | 1.000 | -1.092~  1.258 |  | 7.17 (3.35) | 7.58 (3.42) | 7.33 (3.50) |  | 0.629 | -1.229~  0.465 | 1.000 | -1.138~  0.8.5 | 1.000 | -0.677~  1.177 |  | 2.858 | 0.068 |
| FMA−LE | 17.83 (3.97) | 19.25 (3.70) | 18.42 (4.08) |  | 0.023 | -2.642~  -0.191 | 0.716 | -1.903~  0.737 | 0.412 | -0.633~  2.299 |  | 18.58 (4.58) | 18.75 (4.35) | 18.58 (4.19) |  | 1.000 | -1.074~  0.741 | 1.000 | -1.041~  1.041 | 1.000 | -0.303~  0.637 |  | 2.482 | 0.095 |
| FMA−TOTAL | 52.50 (16.20) | 54.50 (16.27) | 53.75 (16.43) |  | 0.032 | -3.837~  -0.163 | 0.789 | -4.239~  1.739 | 1.000 | -1.582~  3.082 |  | 52.33 (22.03) | 52.25 (22.00) | 52.17 (21.29) |  | 1.000 | -0.799~  0.965 | 1.000 | -1.631~  1.965 | 1.000 | -1.487~  1.653 |  | 4.199 | 0.029^†^ |
| K-MBI | 84.67 (6.81) | 85.17 (6.66) | 85.75 (7.02) |  | 0.498 | -1.451~  0.451 | 0.123 | -2.403~  0.237 | 0.512 | -1.706~  0.539 |  | 82.17 (11.54) | 82.17 (11.54) | 82.17 (11.54) |  | - | - | - | - | - | - |  | 3.591 | 0.036^†^ |

Continuous values are presented as means (standard deviations).

Real HD-tDCS group, robot-assisted gait training with real high-definition transcranial direct current stimulation; Sham HD-tDCS group, robot-assisted gait training with sham high-definition transcranial direct current stimulation; Pre, pre-intervention; Post, post-intervention; F/U, one-month follow-up; 10MWT; 10 Meter Walk Test; TUG, Timed Up and Go; FAC, Functional Ambulation Category; FRT, Functional Reach Test; BBS, Berg Balance Scale; DGI, Dynamic Gait Index; FMA, Fugl-Meyer Assessment; LE, lower extremity; K-MBI, Korean Version of the Modified Barthel Index; 95% CI; 95% Confidence Interval for Difference.

^*^Significant change compared with Pre (*P* < 0.05); ^**^Significant change compared with Pre (*P* < 0.01); ^†^Significant (*P* < 0.05) interaction effects (time × group).

Supplementary Table S3. Stroke lesions in subjects

| Patient number | Group | Stroke lesion |
| --- | --- | --- |
| 1 | Real HD-tDCS group | Rt. thalamic ICH/IVH |
| 2 | Sham HD-tDCS group | Lt. MCA infarction |
| 3 | Real HD-tDCS group | Lt. thalamic ICH/IVH |
| 4 | Sham HD-tDCS group | Lt. ACA, MCA infarction |
| 5 | Real HD-tDCS group | Lt. pons infarction |
| 6 | Real HD-tDCS group | Lt. pons infarction |
| 7 | Sham HD-tDCS group | Rt. MCA infarction |
| 8 | Real HD-tDCS group | Lt. BG ICH |
| 9 | Real HD-tDCS group | Lt. thalamic ICH/IVH |
| 10 | Sham HD-tDCS group | Rt. CR infarction |
| 11 | Sham HD-tDCS group | Lt. pons infarction |
| 12 | Sham HD-tDCS group | Rt. BG ICH |
| 13 | Sham HD-tDCS group | Lt. BG ICH |
| 14 | Real HD-tDCS group | Rt. BG infarction |
| 15 | Real HD-tDCS group | Rt. CR infarction |
| 16 | Real HD-tDCS group | Rt. BG and CR infarction |
| 17 | Sham HD-tDCS group | Rt. MCA infarction |
| 18 | Sham HD-tDCS group | Lt. BG ICH |
| 19 | Real HD-tDCS group | Rt. thalamic infarction |
| 20 | Sham HD-tDCS group | Lt. MCA infarction |
| 21 | Sham HD-tDCS group | Lt. pons infarction |
| 22 | Real HD-tDCS group | Lt. BG ICH |
| 23 | Real HD-tDCS group | Rt. thalamic infarction |
| 24 | Sham HD-tDCS group | Lt. BG and CR infarction |

Real HD-tDCS group, robot-assisted gait training with real high-definition transcranial direct current stimulation; Sham HD-tDCS group, robot-assisted gait training with sham high-definition transcranial direct current stimulation; ICH, Intracerebral Hemorrhage; IVH, Intraventricular Hemorrhage; MCA, Middle Cerebral Artery; ACA, Anterior Cerebral Artery; BG, Basal Ganglia; CR, Corona Radiata.
